# Exercise interventions are most consistently supported for depressive disorders: an umbrella review of diagnosed depressive and anxiety disorders

**DOI:** 10.3389/fpsyt.2026.1871522

**Published:** 2026-07-03

**Authors:** Yafei Gao, Jiayi Zhang, Jingshi Ma, Zikun Lyu

**Affiliations:** Department of Physical Education, Kunsan National University, Gunsan, Republic of Korea

**Keywords:** acceptability, AMSTAR 2, anxiety, corrected covered area, depression, exercise, systematic review, umbrella review

## Abstract

**Background:**

Exercise is increasingly discussed as part of lifestyle-based and multimodal care for mood and anxiety disorders, but review-level evidence often mixes formally diagnosed clinical populations with symptom-defined or medically mixed samples.

**Methods:**

We conducted an umbrella review of systematic reviews, meta-analyses, and network meta-analyses of structured exercise interventions for adults with depressive or anxiety disorders. Six databases were searched from inception to 1 March 2026. Primary outcomes were depressive and anxiety symptom severity, remission, and response; secondary outcomes were acceptability and tolerability. Review quality was appraised with AMSTAR 2, and primary-study overlap was quantified with corrected covered area (CCA), including overall and symptom-cluster analyses. The synthesis was designed to summarize review-level credibility and clinical interpretability rather than to generate a second-order pooled efficacy estimate.

**Results:**

Nine reviews met eligibility criteria; four supplied directly extractable primary overall review-level estimates for core psychiatric symptom outcomes. AMSTAR 2 appraisal rated one review as high, three as low, and five as critically low. Recalculated overall overlap was slight (112 primary-study occurrences, 89 unique primary studies; CCA = 3.23%), although cluster-level analyses identified localized redundancy, particularly within anxiety-disorder-specific reviews. In major depressive disorder, one clinically focused review reported a large reduction in depressive symptoms for aerobic exercise versus non-exercise comparators (Hedges’ g = -0.79, 95% CI -1.00 to -0.57; I² = 21%). Across diagnosed depressive and/or anxiety disorders, broader review-level estimates also favored exercise for depressive symptoms (SMD = -0.97, 95% CI -1.28 to -0.66) and anxiety symptoms (SMD = -0.66, 95% CI -1.09 to -0.23), but heterogeneity was high. Anxiety-disorder-specific evidence was less secure: the primary DSM-IV anxiety-disorder pooled estimate showed no clear benefit over selected controls (SMD = 0.02, 95% CI -0.20 to 0.24). Acceptability estimates were close to null, and adverse-event reporting was too sparse to support confident safety conclusions.

**Conclusion:**

Exercise is best supported as an adjunctive, patient-centered component of care for depressive disorders. Anxiety-disorder-specific efficacy remains uncertain when comparator rigor, diagnostic heterogeneity, and localized overlap are considered, and safety reporting needs substantial improvement.

**Systematic Review Registration:**

https://www.crd.york.ac.uk/PROSPERO/, identifier CRD420261364264.

## Introduction

Depressive and anxiety disorders are among the leading contributors to years lived with disability worldwide, and their burden increased further during the COVID-19 pandemic (1, 2). Pharmacotherapy and psychotherapy remain core evidence-based treatments for mood and anxiety disorders, with extensive comparative evidence supporting antidepressant medication and major psychotherapeutic approaches (3, 4). Contemporary psychiatric guidance increasingly positions lifestyle strategies within broader multimodal care rather than as isolated alternatives (5–7). Exercise is therefore clinically attractive because it is scalable in principle, has broad physical-health benefits, and may be acceptable to patients who prefer non-pharmacological strategies or face cost, access, residual-symptom, or comorbidity barriers.

Earlier meta-analyses of primary trials generally concluded that exercise reduces depressive symptoms, but the magnitude of benefit varies according to trial quality, comparator choice, diagnostic stringency, intervention supervision, and publication bias (8–10). Anxiety-disorder evidence has been more difficult to interpret. Some syntheses found anxiolytic effects, yet anxiety-disorder-specific findings were often sensitive to waitlist, placebo, or otherwise weak comparator designs (11–14). A recent broad overview of physical activity interventions reported benefits for depression, anxiety, and distress across adult populations, but the underlying evidence base included non-clinical, symptom-defined, medically mixed, and stress-related samples that are not interchangeable with formally diagnosed psychiatric disorders (15).

Review-level evidence relevant to clinically diagnosed depressive and anxiety disorders has continued to accumulate, including aerobic exercise in major depressive disorder, outpatient exercise and mind-body interventions, yoga, web-based exercise, traditional Chinese exercise, panic-disorder-focused evidence, and broader diagnosed depression/anxiety exercise syntheses (16–22). These reviews differ materially in diagnostic boundaries, eligible comparators, intervention modalities, and whether they present a directly comparable pooled overall estimate. Such heterogeneity matters for psychiatric interpretation because the claim that exercise improves depressive or anxiety symptoms in mixed populations is not equivalent to the claim that exercise is a reliable treatment component for formally diagnosed mood or anxiety disorders.

We therefore conducted an umbrella review to synthesize the efficacy, acceptability, and tolerability of exercise interventions for adults with depressive or anxiety disorders. The review was designed to fit a clinically oriented psychiatry readership by prioritizing formally diagnosed populations, distinguishing depressive-disorder from anxiety-disorder evidence, separating directly extractable review-level pooled estimates from narrative-only evidence, and incorporating methodological appraisal, diagnostic-rigor assessment, and primary-study overlap into interpretation.

## Methods

### Design and registration

This umbrella review synthesized systematic reviews, meta-analyses, and network meta-analyses of exercise interventions for depressive and anxiety disorders. Reporting followed PRISMA 2020 guidance, and the protocol was prospectively registered in PROSPERO (CRD420261364264) (23). The umbrella-review approach followed established methods for summarizing systematic reviews while using reviews, rather than primary trials, as the unit of analysis (24). Because eligible reviews differed in diagnostic boundaries, comparator frameworks, intervention classes, effect metrics, and publication-bias handling, the primary aim was to evaluate review-level credibility and clinically interpretable patterns of evidence rather than to derive a single unified pooled treatment-effect estimate.

### Eligibility criteria and PICOS

The population of interest was adults with depressive disorders or anxiety disorders. Formal diagnostic frameworks, including DSM, ICD, MINI, SCID, or equivalent clinical diagnostic procedures, were preferred and used to define the core target population wherever available. For the core diagnosed-disorder interpretation, reviews were expected to require formal psychiatric diagnosis in all or nearly all primary studies, or to document that at least 75% of included primary studies or participants involved formally diagnosed, treatment-seeking, or otherwise clearly clinical psychiatric samples. Reviews that targeted depressive or anxiety disorders but used mixed diagnostic and validated symptom-threshold criteria, or did not report sufficient primary-study diagnostic proportions, were retained only as indirect narrative or supplementary evidence and were not allowed to drive the principal diagnosed-disorder conclusions. Mixed psychiatric-medical populations were eligible only when depressive or anxiety disorders were the target condition or a disorder-specific psychiatric subgroup was extractable; reviews focused primarily on medical populations with secondary mood or anxiety symptoms were excluded. Reviews focused only on elevated symptoms, subthreshold symptoms, healthy populations, children or adolescents, or conditions outside the predefined boundary, such as post-traumatic stress disorder, obsessive-compulsive disorder, or acute stress disorder, were excluded. These exclusions were applied because these conditions have distinct diagnostic and treatment frameworks and would have broadened the psychiatric scope beyond the prespecified depressive- and anxiety-disorder question. Interventions were structured exercise programs, including aerobic exercise, resistance exercise, mixed aerobic-plus-resistance programs, yoga, tai chi, qigong, baduanjin, wuqinxi, and web-based exercise delivery. Comparators included usual care, inactive control, no intervention, waitlist, placebo, active control, pharmacotherapy, psychotherapy, and head-to-head exercise comparisons. Outcomes were depressive symptom severity, anxiety symptom severity, remission, response, dropout, discontinuation, attendance, adherence, completion, and adverse events. Eligible study designs were systematic reviews, meta-analyses, and network meta-analyses.

### Information sources and search strategy

We searched PubMed, Web of Science, SPORTDiscus, Cochrane Library, Embase, and Scopus from inception to 1 March 2026. Search concepts combined exercise terms, depressive and anxiety disorder terms, and review or meta-analysis filters. Database-specific strategies are provided in **Supplementary Table 1**. Reference lists of eligible reviews were also checked.

### Study selection and data extraction

Search results were deduplicated and screened against predefined eligibility criteria. Full texts of potentially relevant records were reviewed for eligibility. For each included review, we extracted bibliographic details, review type, search date coverage, target disorder, diagnostic framework, diagnostic-rigor stratum, intervention and comparator scope, primary-study designs, sample size, pooled effect estimates, confidence intervals, heterogeneity statistics, publication-bias and sensitivity findings, acceptability outcomes, and methodological-quality information. Extraction retained the source-reported metric, effect direction, confidence interval, and heterogeneity statistic rather than forcing conversions across metrics. Diagnostic rigor and indirectness judgments are reported in **Supplementary Table 16**.

### Methodological appraisal and primary-study overlap

Methodological quality was appraised with AMSTAR 2, retaining item-level judgments and overall confidence ratings (25). Because umbrella reviews are vulnerable to double counting when multiple reviews contain the same primary trials, overlap was assessed using a normalized review-by-primary-study citation matrix and summarized with corrected covered area (CCA) (26). CCA was calculated from auditable primary-study entries; non-trial-level or otherwise internally inconsistent overlap summaries were not used for manuscript interpretation. To assess localized redundancy that could be obscured by the overall matrix, we also calculated subset CCAs restricted to the reviews contributing to the core depressive-symptom and anxiety-symptom evidence clusters, with a sensitivity calculation for anxiety-disorder-specific reviews.

### Synthesis strategy

We did not re-pool primary-study data or perform a second-order umbrella meta-analysis because comparator frameworks, diagnostic boundaries, intervention classes, effect metrics, and sign conventions differed materially across reviews. Instead, we used structured narrative synthesis supported by review-level tables and figures. The synthesis was therefore a credibility and clinical-interpretability map of source reviews, not a unified quantitative treatment-effect model. When multiple pooled analyses were available within a review, the prespecified hierarchy prioritized primary overall pooled estimates for core psychiatric symptom outcomes over subgroup, sensitivity, publication-bias-adjusted, and non-core secondary estimates. For cross-review plotting only, effect directions were harmonized so that positive values uniformly favored exercise; source-reported estimates and harmonization decisions are documented in the **Supplementary Appendix**. Acceptability and tolerability were synthesized separately because outcomes and metrics were sparse and heterogeneous.

## Results

### Study selection

**Figure 1:**
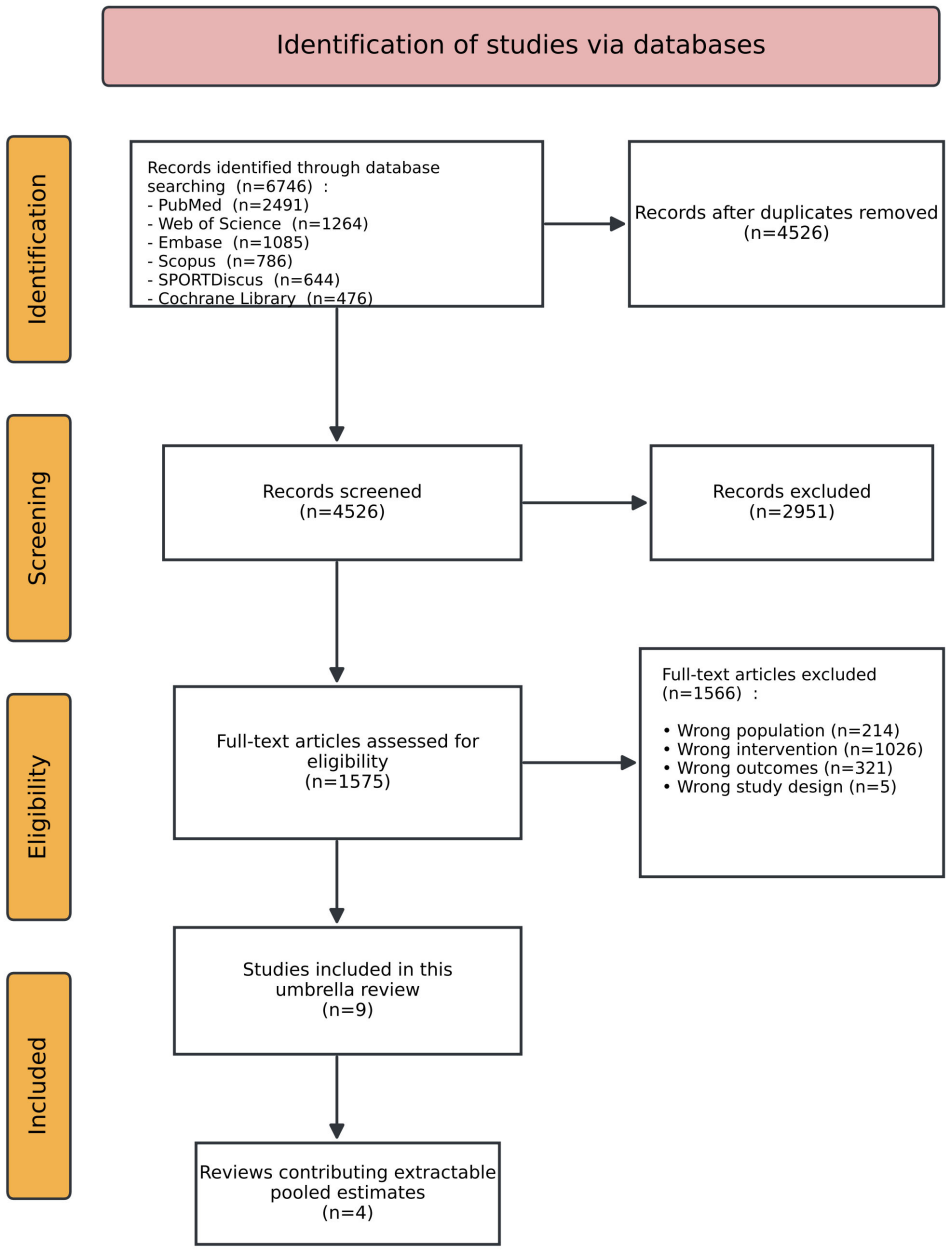
PRISMA flow diagram for the umbrella review.

The database search identified 6,746 records. After removal of 2,220 duplicates, 4,526 records underwent title and abstract screening; 2,951 were excluded. We assessed 1,575 full-text articles and excluded 1,566 records: wrong population (n = 214), wrong intervention (n = 1,026), wrong outcomes (n = 321), and wrong study design (n = 5). Nine reviews met inclusion criteria, and four supplied source-reported, extractable primary overall pooled estimates for core psychiatric symptom outcomes at the review level; these estimates were displayed descriptively and were not statistically combined across reviews. The study-selection process is shown in **Figure 1**.

### Characteristics of included reviews

The nine included reviews were published between 2013 and 2025 and covered clinically diagnosed depressive disorders, anxiety disorders, or mixed diagnosed depressive and/or anxiety samples (**Table 1**). Four reviews focused primarily on depressive disorders, three on anxiety disorders, and two on broader diagnosed depressive and/or anxiety samples. The anxiety evidence was unevenly distributed across diagnostic categories: one review pooled DSM-IV anxiety disorders, one synthesized clinical anxiety disorders narratively, one focused specifically on panic disorder with or without agoraphobia, and broader mixed-disorder reviews did not isolate generalized anxiety disorder or social anxiety disorder in separate pooled estimates. Intervention scope ranged from aerobic exercise alone to aerobic, resistance, and mixed exercise, yoga, tai chi, qigong, baduanjin, wuqinxi, and web-based exercise delivery. Five reviews reported some quantitative synthesis of core psychiatric outcomes, but only four supplied a source-reported, directly extractable primary overall pooled estimate suitable for descriptive cross-review display in the main review-level efficacy figure. Diagnostic-rigor strata and indirectness judgments are provided in **Supplementary Table 16**.

**Table 1:** Characteristics of included systematic reviews and meta-analyses.

Review	Design	Disorder focus	Diagnostic boundary	Intervention	Comparator	Included studies/sample	Acceptability/tolerability
Banyard et al. (22)	Meta-analysis	Diagnosed depression and/or anxiety	Adults 18–64 years with diagnosed depression and/or anxiety	Aerobic, resistance, or mixed exercise; yoga excluded	Non-exercise/usual care	32 RCTs; 3,243 participants	Attendance/adherence
Bartley et al. (11)	Meta-analysis	DSM-IV anxiety disorders	Primary DSM-defined anxiety disorder; PTSD excluded	Aerobic exercise	Waitlist/placebo, non-aerobic exercise, CBT, psychoeducation/meditation, medication comparison	7 RCTs; 407 participants	No pooled acceptability
Carneiro et al. (19)	Systematic review	Depressive and/or anxiety disorders or above-threshold symptoms	DSM/ICD diagnosis or validated symptom thresholds; formal-diagnosis proportion not consistently auditable	Web-, email-, or app-based home exercise	Active/passive controls	Dropout/adverse events narrative; indirect for core diagnosed-disorder conclusions	Dropout/adverse events narrative
Cramer et al. (18)	Systematic review	Major depressive disorder	DSM-IV/V MDD; *post-hoc* inclusion if ≥75% MDD	Yoga	No treatment, attention control, pharmacological, non-pharmacological, aerobic exercise, ECT	7 RCTs; 240 participants	Adverse events in 2 RCTs
Feng et al. (20)	Network meta-analysis	Depression	Adults meeting diagnostic criteria for depression	Baduanjin, Tai Chi, Wuqinxi ± medication	Routine medication/conventional treatment or blank control	25 RCTs; 1,605 participants	No
Jayakody et al. (12)	Systematic review	Clinical anxiety disorders	Formal ICD/DSM/validated anxiety-disorder diagnosis	Exercise alone or adjunctive	Medication, CBT, educational/GP care, relaxation, placebo/no intervention, active exercise	8 RCTs; 563 participants	Compliance narrative
Machado et al. (21)	Systematic review	Panic disorder	DSM/ICD/MINI panic disorder with/without agoraphobia	Regular aerobic, strength, or multimodal exercise	Active/waiting controls, usual care, psychotherapy, pharmacotherapy	8 trials; 352 participants	Dropout/adherence narrative
Morres et al. (16)	Meta-analysis	Major depressive disorder	Adults recruited through mental health services with clinical MDD diagnosis	Aerobic exercise	Any non-exercise comparator	11 trials/13 comparisons; 455 participants	Dropout meta-analysis
Seshadri et al. (17)	Meta-analysis	Outpatient major depressive disorder	Current-episode MDD, ICD-10 or DSM-IV/5, ≥8 weeks follow-up	Exercise, yoga, and tai chi	Any control condition	25 RCTs; 2,083 participants	No dedicated pooled acceptability

RCT, randomized controlled trial; MDD, major depressive disorder; CBT, cognitive behavioral therapy; ECT, electroconvulsive therapy; TAU, treatment as usual.

### Methodological quality and primary-study overlap

**Figure 2:**
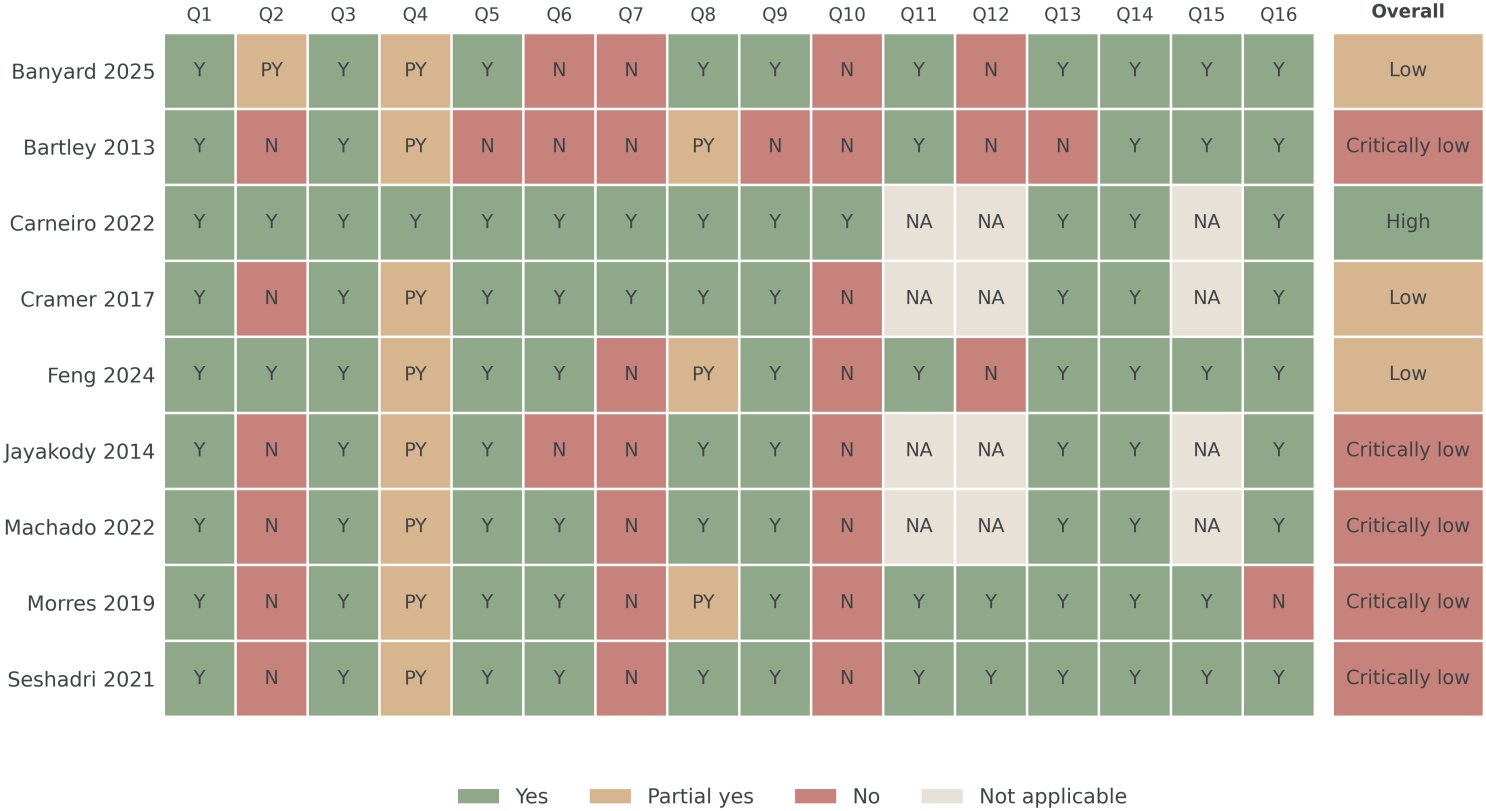
AMSTAR 2 item-level appraisal of the included reviews. Y, yes; PY, partial yes; N, no; NA, not applicable. The overall confidence column reflects the AMSTAR 2 reanalysis.

**Figure 3:**
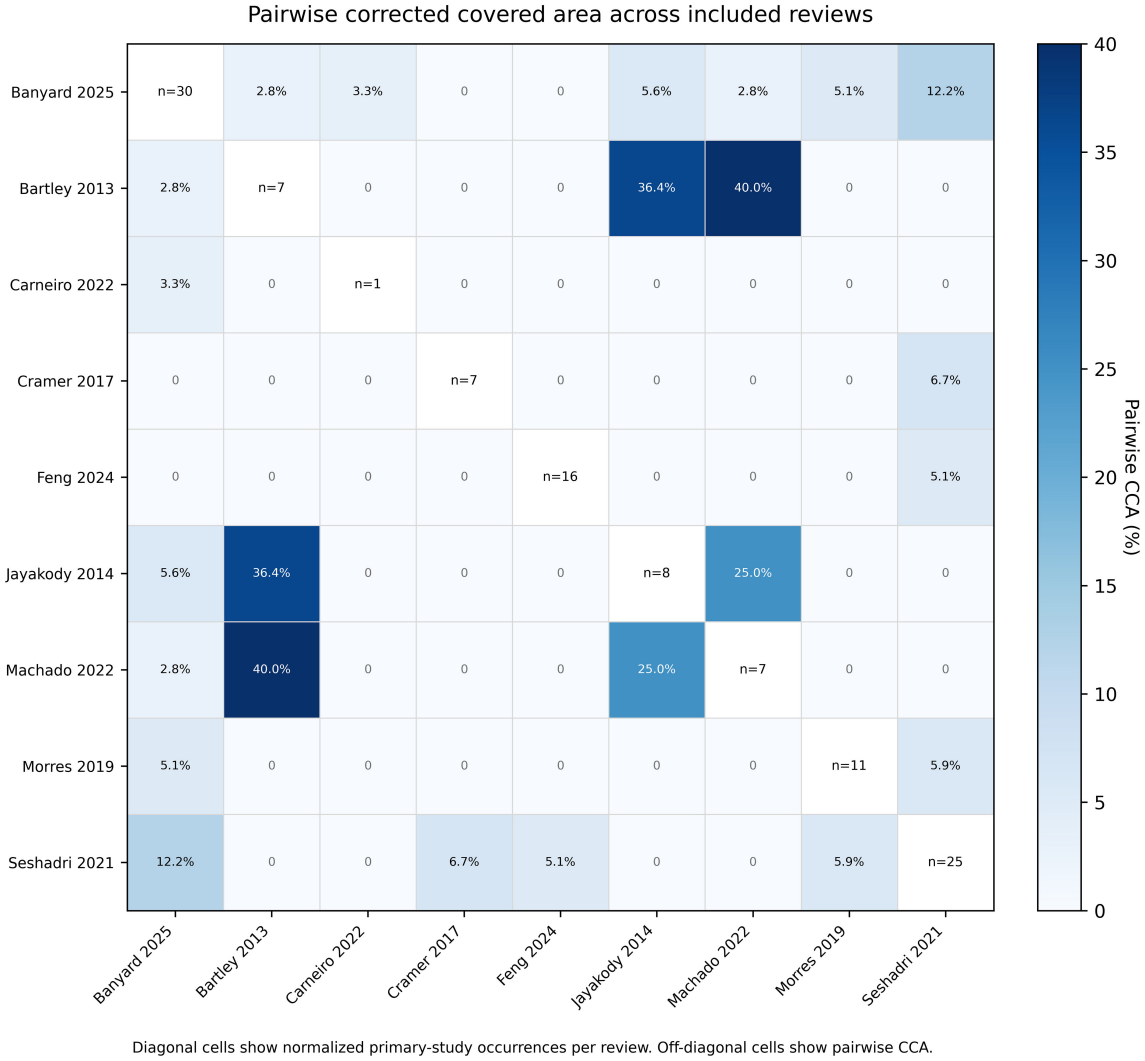
Pairwise corrected covered area heatmap across included reviews. Off-diagonal cells represent pairwise CCA values derived from the normalized review-by-primary-study citation matrix; diagonal cells display the number of normalized primary-study occurrences within each review. The normalized citation matrix contained 112 primary-study occurrences and 89 unique primary studies across nine reviews, giving an overall CCA of 3.23%. Cluster-level subset CCA values are reported in **Supplementary Table 17**.

AMSTAR 2 appraisal rated one review as high confidence, three as low confidence, and five as critically low confidence (**Table 2**; **Figure 2**). Common weaknesses included absent or incomplete protocol reporting, incomplete lists or justifications for excluded studies, incomplete reporting of primary-study funding, and variable integration of primary-study risk of bias into interpretation. The recalculated normalized citation matrix contained 112 primary-study occurrences, 89 unique primary studies, and nine reviews, yielding a slight overall CCA of 3.23%. Cluster-specific CCA analyses showed greater localized redundancy than the overall value: the core depression quantitative cluster (Morres 2019, Seshadri 2021, and Banyard 2025) had moderate overlap (66 occurrences, 58 unique primary studies; CCA = 6.90%); the two reviews contributing directly to the plotted quantitative anxiety estimates (Bartley 2013 and Banyard 2025) had slight overlap (37 occurrences, 36 unique primary studies; CCA = 2.78%); and the broader anxiety symptom cluster that also included narrative anxiety reviews (Bartley 2013, Jayakody 2014, Machado 2022, and Banyard 2025) had moderate overlap (52 occurrences, 42 unique primary studies; CCA = 7.94%). A sensitivity analysis restricted to the three anxiety-disorder-specific reviews showed very high overlap (22 occurrences, 14 unique primary studies; CCA = 28.57%), driven by duplicated panic/anxiety trials. Because no second-order meta-analysis was performed, these localized overlaps were used to qualify certainty rather than to assign pooled weights. Pairwise overlap was concentrated in related evidence families, with the largest shared-study counts between Banyard 2025 and Seshadri 2021 (n = 6), Bartley 2013 and Jayakody 2014 (n = 4), and Bartley 2013 and Machado 2022 (n = 4). The pairwise CCA heatmap is shown in **Figure 3**, and subset CCA analyses are reported in **Supplementary Table 17**.

**Table 2:** AMSTAR 2 methodological quality and primary-study overlap summary.

Review	AMSTAR 2 overall	Main methodological limitations	Primary-study entries	Unique to review	Maximum pairwise overlap	Unique contribution
Banyard 2025	Low	Partial protocol/search reporting; no excluded-study list; incomplete funding/COI reporting	30	21	6 with Seshadri 2021	Broadest diagnosed depression/anxiety exercise-mode review
Bartley 2013	Critically low	No protocol; partial search; no excluded-study list; limited RoB integration	7	2	4 with Jayakody 2014 and Machado 2022	Only pooled DSM-IV anxiety-disorder aerobic-exercise meta-analysis
Carneiro 2022	High	Meta-analysis not applicable because only three heterogeneous RCTs	1	0	1 with Banyard 2025	Only web-based exercise review in strict set
Cramer 2017	Low	No protocol; partial search details; no meta-analysis	7	5	2 with Seshadri 2021	Yoga-specific MDD review
Feng 2024	Low	Partial search; no excluded-study list; incomplete source funding handling	16	14	2 with Seshadri 2021	Traditional Chinese exercise network meta-analysis
Jayakody 2014	Critically low	No protocol; no excluded-study list; incomplete duplicate processes and funding reporting	8	3	4 with Bartley 2013	Comparison-specific anxiety-disorder review
Machado 2022	Critically low	No protocol; no excluded-study list; incomplete funding reporting; no pooled synthesis	7	3	4 with Bartley 2013	Panic-disorder-specific exercise review
Morres 2019	Critically low	No protocol; partial search; no excluded-study list; incomplete source funding/COI reporting	11	9	2 with Banyard 2025 and Seshadri 2021	Most clinically strict AE-in-MDD mental-health-services meta-analysis
Seshadri 2021	Critically low	No protocol; partial search; no excluded-study list; incomplete source funding reporting	25	15	6 with Banyard 2025	Comprehensive outpatient MDD review spanning exercise/yoga/tai chi

Overall corrected covered area from the normalized citation matrix was 3.23%, indicating slight overlap overall. Pairwise overlap is reported as shared primary-study entries rather than as a second-order pooled weight; cluster-specific CCA values are reported in **Supplementary Table 17**.

### Depressive-disorder outcomes

**Figure 4:**
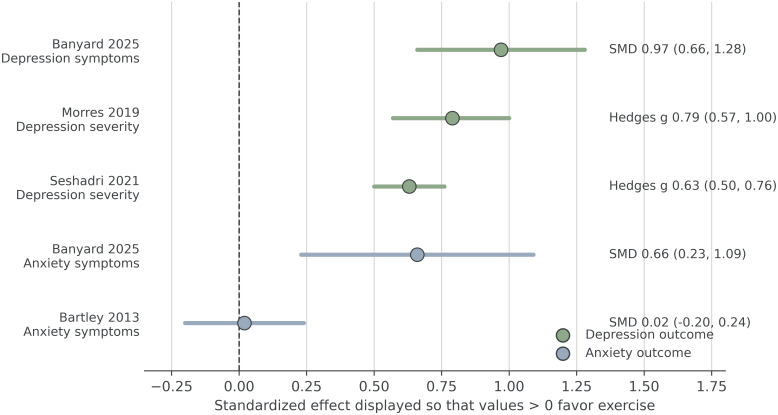
Primary overall pooled efficacy estimates for core psychiatric symptom outcomes. Values were harmonized for plotting so that estimates greater than 0 favor exercise. Source-reported metrics and original orientations are retained in the **Supplementary Appendix**.

The clearest quantitative signal concerned depressive symptom severity in major depressive disorder (**Table 3** and **Figure 4**). A meta-analysis restricted to adults with major depressive disorder recruited through mental health services reported a large benefit of aerobic exercise versus non-exercise comparators (Hedges’ g = -0.79, 95% CI -1.00 to -0.57; I² = 21%) (16). This source-reported estimate was based on a comparatively narrow diagnostic and setting boundary and was one of the most clinically interpretable depression-specific findings. An outpatient major depressive disorder review combining exercise, yoga, and tai chi also reported a favorable source-reported overall effect (Hedges’ g = 0.63, 95% CI 0.50 to 0.76; I² = 98%) (17). However, the I² of 98% indicates extreme statistical heterogeneity and substantial clinical incoherence across intervention modalities, settings, and comparator conditions; under such conditions, the central pooled estimate has limited clinical interpretability as a single exercise treatment effect. Separately, trim-and-fill adjustment in that review attenuated the estimate to Hedges’ g = 0.11 (95% CI -0.03 to 0.26), which addresses potential small-study or publication-bias effects but does not reduce or resolve the extreme heterogeneity. In a broader review of adults with diagnosed depression and/or anxiety disorders, exercise was associated with lower depressive symptoms (SMD = -0.97, 95% CI -1.28 to -0.66; I² = 90.1%) (22), but the high heterogeneity and mixed diagnostic scope limit confidence in the magnitude. Narrative depressive-disorder reviews suggested that yoga and traditional Chinese exercise may be promising, but the available evidence was small, heterogeneous, or not fully auditable for the main quantitative hierarchy (18, 20).

**Table 3:** Best available efficacy findings by disorder, intervention, comparator, and core outcome.

Population	Exercise intervention	Comparator	Core outcome	Prioritized review	Best available result	95% CI	I²	Interpretation/caveat
MDD	Aerobic exercise	Non-exercise comparators	Depression severity	Morres 2019	Hedges g = -0.79	-1.00 to -0.57	21%	Large source-reported antidepressant signal in clinically diagnosed adults from mental health services; AMSTAR 2 critically low.
Outpatient MDD	Exercise/yoga/tai chi	All controls	Depression severity	Seshadri 2021	Hedges g = 0.63	0.50 to 0.76	98%	Favorable source-reported estimate, but I² = 98% makes the central mean clinically difficult to interpret; trim-and-fill addresses small-study effects separately and attenuated g to 0.11 (-0.03 to 0.26).
MDD	Yoga	Aerobic exercise/imipramine/ECT/attention/medication add-on	Depression/remission/anxiety/safety	Cramer 2017	Narrative	—	—	Some evidence beyond placebo; too few small trials for firm inference.
Diagnosed depression and/or anxiety	Aerobic/resistance/mixed exercise	All controls	Depression symptoms	Banyard 2025	SMD = -0.97	-1.28 to -0.66	90.1%	Large favorable review-level estimate with substantial heterogeneity and mixed diagnostic scope.
Diagnosed depression and/or anxiety	Aerobic/resistance/mixed exercise	All controls	Anxiety symptoms	Banyard 2025	SMD = -0.66	-1.09 to -0.23	85.8%	Moderate favorable review-level estimate, but not anxiety-disorder specific and accompanied by substantial heterogeneity.
DSM-IV anxiety disorders	Aerobic exercise	Selected best time-matched/non-time-matched controls	Anxiety symptoms	Bartley 2013	SMD = 0.02	-0.20 to 0.24	Not reported	No clear difference in DSM-IV anxiety disorders; favorable effects concentrated in waitlist/placebo or time-uncontrolled comparisons.
Clinical anxiety disorders	Exercise	Medication/CBT/GP care/active exercise	Anxiety symptoms	Jayakody 2014	Narrative	—	—	Adjunctive benefit suggested in selected comparisons; direct medication comparison favored antidepressant treatment.
Panic disorder	Regular exercise	Active/waiting controls, CBT, pharmacotherapy	Panic/global anxiety/depression	Machado 2022	Narrative	—	—	No clear panic-specific benefit; global anxiety/depression often improved.
Depressive/anxiety disorders or above-threshold symptoms	Web-based exercise	Active/passive controls	Depressive/anxiety symptoms	Carneiro 2022	Narrative	—	—	Insufficient and diagnostically indirect evidence; no clear anxiety superiority; compliance concerns.
Depression	Baduanjin/Tai Chi/Wuqinxi	Routine/blank controls	HAMD/BDI/SDS/HAMA/PSQI	Feng 2024	Network meta-analysis	See supplement	High across outcomes	Promising but league-table orientation not sufficiently auditable for main quantitative synthesis.

When multiple pooled analyses were available, the primary overall estimate was prioritized over subgroup, sensitivity, publication-bias-adjusted, or non-core secondary analyses. Narrative rows are retained when they materially inform disorder-specific interpretation. HAMD, Hamilton Depression Rating Scale; BDI, Beck Depression Inventory; SDS, Self-rating Depression Scale; HAMA, Hamilton Anxiety Rating Scale; PSQI, Pittsburgh Sleep Quality Index.

### Anxiety-disorder outcomes

Anxiety-disorder-specific evidence was less secure than depressive-disorder evidence (**Table 3**; **Figure 4**). The represented anxiety evidence should not be interpreted as homogeneous: Bartley 2013 pooled DSM-IV anxiety disorders, Jayakody 2014 summarized clinical anxiety disorders across heterogeneous single-study comparisons, Machado 2022 focused on panic disorder with or without agoraphobia, and the broader Banyard 2025 estimate combined diagnosed depression and/or anxiety without isolating anxiety disorders. No included review supplied a disorder-specific pooled estimate for generalized anxiety disorder or social anxiety disorder, and panic disorder was the only separately focused anxiety diagnosis. In the primary pooled analysis of DSM-IV anxiety disorders, aerobic exercise showed no clear benefit over selected controls (SMD = 0.02, 95% CI -0.20 to 0.24), and the review noted that apparently favorable effects were concentrated in waitlist, placebo, or time-uncontrolled comparisons (11). In the broader diagnosed depression and/or anxiety review, exercise was associated with lower anxiety symptoms (SMD = -0.66, 95% CI -1.09 to -0.23; I² = 85.8%) (22), but this estimate did not isolate anxiety disorders and was accompanied by substantial heterogeneity. Narrative anxiety reviews mainly supported adjunctive or context-dependent benefit rather than robust stand-alone superiority; panic-disorder evidence did not clearly support a panic-specific effect despite improvements in global anxiety and depressive symptoms in some studies (12, 21). These findings support only cautious anxiety-related conclusions.

### Direct comparisons, acceptability, and tolerability

Direct evidence against established pharmacotherapy and psychotherapy was sparse and heterogeneous. Available findings did not justify claims that exercise was superior to antidepressant medication or psychotherapy; rather, the pattern was more consistent with exercise as an adjunctive or supportive option in selected contexts. Acceptability, tolerability, and safety outcomes were under-reported relative to symptom outcomes (**Table 4**) and were treated as distinct from efficacy. Quantitative acceptability findings were close to null: attendance did not clearly differ between exercise and controls (RR = 1.11, 95% CI 0.93 to 1.32; I² = 35.9%) (22), and dropout risk difference was near zero for aerobic exercise versus non-exercise comparators in major depressive disorder (RD = 0.01, 95% CI -0.03 to 0.05; I² = 0%) (16). However, attendance and dropout do not fully capture tolerability, symptom worsening, musculoskeletal events, cardiovascular events, or reasons for discontinuation. Adverse-event reporting was sparse and inconsistent, and lack of reported adverse events should not be interpreted as evidence that exercise interventions are uniformly safe. **Supplementary Figure 1** displays the sparse quantitative acceptability outcomes.

**Table 4:** Best available acceptability and tolerability findings.

Population	Intervention	Outcome	Review(s)	Best available result	95% CI/heterogeneity	Interpretation
Diagnosed depression and/or anxiety	Aerobic/resistance/mixed exercise	Attendance	Banyard 2025	RR = 1.11	0.93 to 1.32; I² = 35.9%	Similar attendance; no clear acceptability advantage and no safety inference.
MDD	Aerobic exercise	Dropout risk difference	Morres 2019	RD = 0.01	-0.03 to 0.05; I² = 0%	No meaningful dropout difference; dropout alone does not establish tolerability.
Depressive/anxiety disorders or above-threshold symptoms	Web-based exercise	Compliance/dropout	Carneiro 2022	Narrative	—	Compliance low and dropout notable in a small, diagnostically indirect evidence base.
MDD	Yoga	Treatment-related adverse events	Cramer 2017	Narrative	—	Only two RCTs assessed treatment-related adverse events; absence of reported events is not evidence of comprehensive safety.
Clinical anxiety disorders/panic disorder	Exercise	Compliance/dropout	Jayakody 2014; Machado 2022	Narrative	—	Sparse and non-pooled reporting; no robust acceptability or safety conclusion.

Acceptability and tolerability outcomes were too sparse and heterogeneous for umbrella-level pooling. RD, risk difference; RR, risk ratio.

### Evidence overview

**Figure 5:**
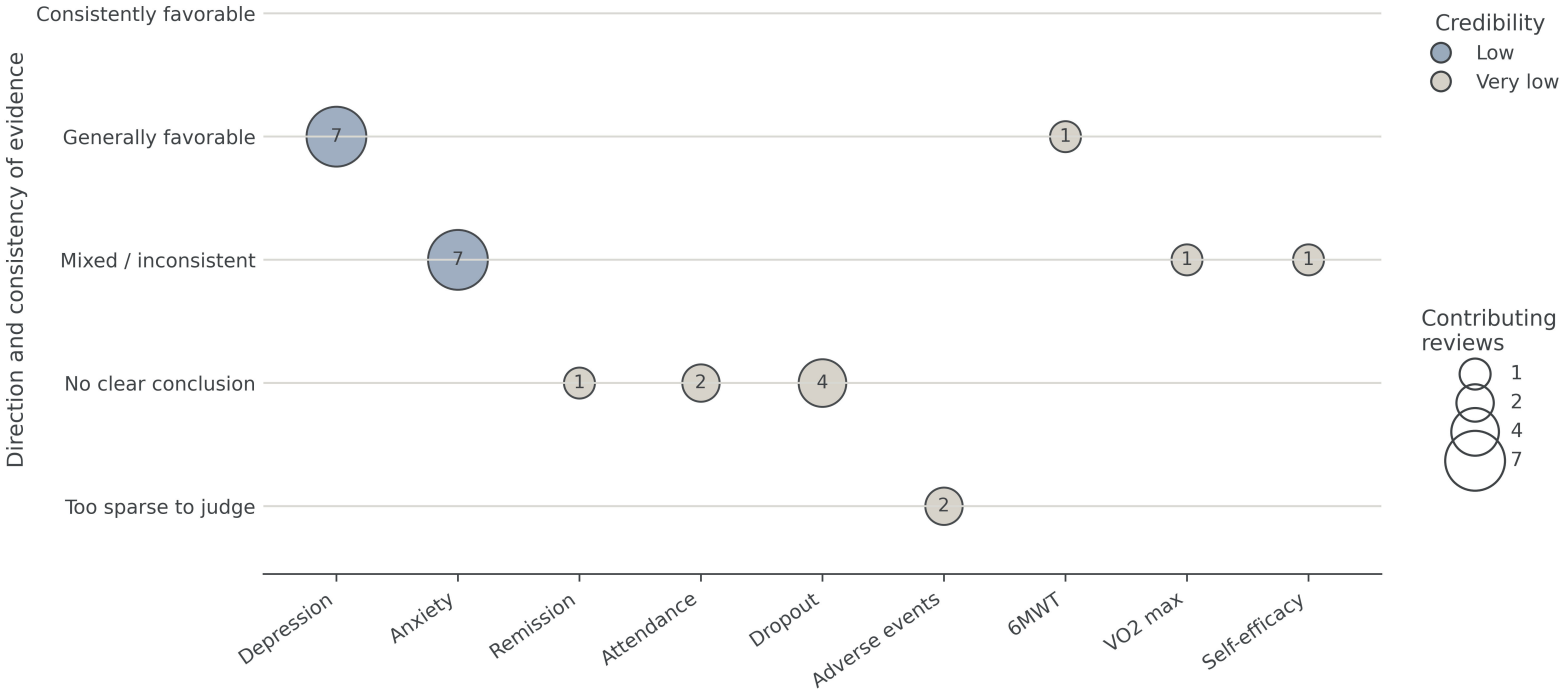
Evidence-overview bubble plot. The vertical axis summarizes the direction and consistency of evidence, bubble size represents the number of contributing reviews, and color represents credibility rating. Non-core outcomes are shown to contextualize the evidence base but were not used to drive the main psychiatric efficacy conclusion.

The evidence-overview bubble plot integrates direction, credibility, and the number of contributing reviews across core and secondary outcomes (**Figure 5**). Depression symptom severity was generally favorable but low in credibility because review quality, heterogeneity, publication-bias concerns, and localized overlap limited certainty. Anxiety symptom severity was mixed or inconsistent despite a favorable broader mixed-disorder estimate, largely because anxiety-disorder-specific evidence was comparator-sensitive, diagnostically uneven, and locally redundant across anxiety-specific reviews. Remission/response, adherence, dropout, and adverse-event outcomes were very low in credibility because they were sparsely and inconsistently reported. Non-core outcomes such as six-minute walk test, VO2 max, and self-efficacy were retained in the **Supplementary Materials** and should not drive the psychiatric efficacy conclusion.

## Discussion

### Main findings

**Figure 6:**
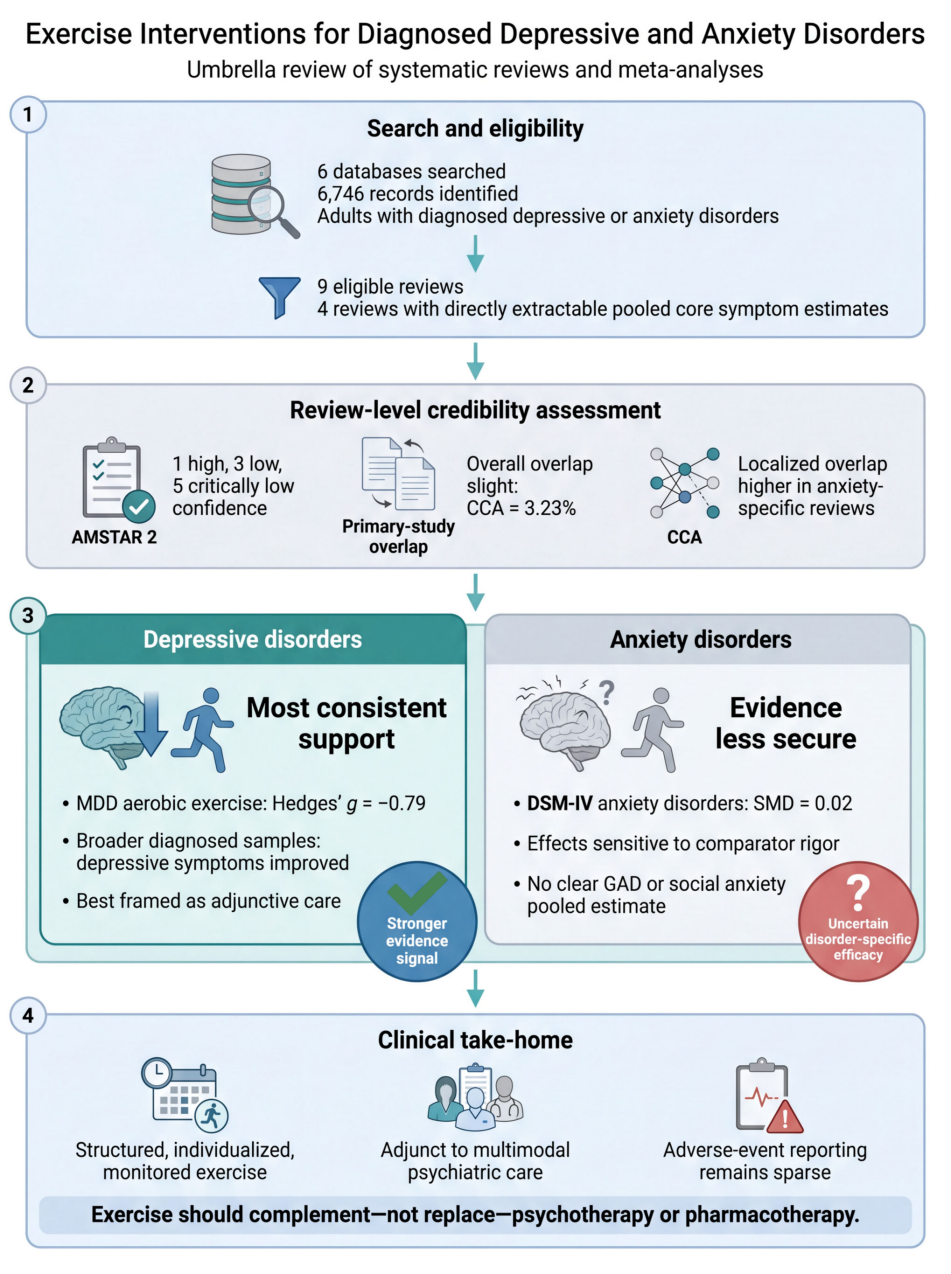
Graphical abstract of the umbrella review of exercise interventions for diagnosed depressive and anxiety disorders. Evidence was most consistent for depressive symptom reduction, particularly in major depressive disorder, whereas anxiety-disorder-specific efficacy and safety conclusions remained less certain. Exercise should be considered a structured adjunct to multimodal psychiatric care. Created with BioRender.com.

This umbrella review indicates that exercise interventions are most consistently supported for depressive symptom reduction in adults with formally diagnosed depressive disorders, especially major depressive disorder. The most clinically stringent signal came from aerobic exercise in adults with major depressive disorder recruited through mental health services, while broader diagnosed samples also showed favorable depressive-symptom estimates. However, these are source-reported review-level estimates rather than results of a unified second-order meta-analysis. In contrast, anxiety-disorder-specific evidence remained weaker, diagnostically uneven, more sensitive to comparator rigor, and locally redundant across anxiety-focused reviews. AMSTAR 2 appraisal and the evidence-overview plot temper the headline effects: most reviews were low or critically low in confidence, and favorable effects were often accompanied by heterogeneity, small-study concerns, or publication-bias signals. Overall CCA was slight, but cluster-specific analyses showed moderate overlap in the core depression and anxiety symptom clusters and very high overlap among anxiety-disorder-specific reviews. This pattern reduces concern about broad ecosystem-wide duplicate counting but supports caution when interpreting anxiety-specific conclusions. A graphical summary of the review-level evidence structure and clinically cautious interpretation is provided in **Figure 6**.

### Relation to broader evidence

The depression-specific pattern aligns with broader lifestyle psychiatry, physical activity, and recent primary-trial synthesis literature showing that exercise-related interventions can improve mental-health symptoms (6, 15, 27–29). It is also directionally consistent with prospective evidence linking physical activity or cardiorespiratory fitness to lower later risk of depression and anxiety (30–33). However, those literatures answer partly different questions. They frequently combine formally diagnosed samples with elevated-symptom, non-clinical, chronic-disease, or medically mixed populations. Our stricter psychiatric boundary yielded a smaller and less uniform evidence base, particularly for anxiety disorders. This distinction is important for Frontiers in Psychiatry readers because prevention, general symptom improvement, and treatment of diagnosed disorders are related but not interchangeable claims.

### Interpretation of depressive-disorder findings

The antidepressant signal is consistent with earlier primary-trial meta-analyses showing that exercise can reduce depressive symptoms (8–10, 34–36). The more clinically focused evidence in this umbrella review suggests that structured aerobic exercise can be a credible adjunctive option for major depressive disorder. Yet the confidence placed in the magnitude of benefit should remain cautious. The outpatient MDD estimate combined exercise, yoga, and tai chi and had extreme heterogeneity, while the trim-and-fill analysis suggested a separate small-study or publication-bias concern. Other reviews contained modality, setting, dose, supervision, comparator, and baseline-severity heterogeneity. These findings support a pragmatic interpretation: exercise may offer clinically meaningful benefit when delivered with adequate structure, support, and monitoring, but the available review-level evidence should not be used to imply a uniform effect across all exercise modes, intensities, settings, or patient profiles.

### Heterogeneity and effect-size interpretation

Clinical heterogeneity and statistical heterogeneity were interpreted as related but distinct threats. Clinical heterogeneity reflected differences in exercise modality, dose, supervision, treatment setting, comparator rigor, baseline symptom severity, co-interventions, and review methodology. Statistical heterogeneity quantified inconsistency in observed effects within a source meta-analysis but did not identify which clinical factors caused it. Publication-bias or trim-and-fill analyses address potential small-study effects; they do not make clinically disparate studies comparable and do not reduce I². Therefore, when heterogeneity was extreme, the central pooled estimate was treated as a fragile summary of a diverse evidence set rather than as a directly generalizable treatment effect. This approach also reduces the risk of overstating efficacy, because large standardized effects from heterogeneous and lower-quality reviews may overestimate the effect likely to be achieved in routine psychiatric care.

### Why anxiety findings were less consistent

Anxiety findings appeared especially sensitive to comparator design and diagnostic composition. The main DSM-IV anxiety-disorder meta-analysis showed no clear advantage for aerobic exercise over selected controls, and favorable effects were concentrated in weaker comparator frameworks (11). Broader anxiety-related reviews have reported anxiolytic effects of exercise, including across anxiety and stress-related conditions and clinical anxiety presentations (13, 14, 37–39), but those syntheses used wider diagnostic frames than the present umbrella review. The present anxiety evidence was concentrated in mixed anxiety presentations and panic-disorder-focused evidence, with no disorder-specific pooled estimates for generalized anxiety disorder or social anxiety disorder. Panic disorder may have plausible exercise-related mechanisms through interoceptive exposure, physical-conditioning, or reduced fear of bodily sensations, but the available review-level evidence did not show a clear panic-specific effect. We excluded PTSD, OCD, and acute stress disorder because their diagnostic frameworks, index symptoms, and treatment comparators differ from the prespecified depressive- and anxiety-disorder scope; including them would have broadened the question and further increased indirectness. The current evidence therefore supports careful clinical framing: exercise may help some patients with anxiety symptoms, particularly as an adjunctive strategy or as part of broader behavioral activation and physical-health support, but the disorder-specific efficacy signal remains uncertain when active and time-/attention-matched controls are handled rigorously.

### Mechanistic and implementation considerations

Exercise interventions may influence depressive and anxiety symptoms through overlapping biological, psychological, and social pathways, including improved sleep, reduced inflammation, enhanced self-efficacy, behavioral activation, social contact, improved physical function, and greater cardiorespiratory fitness. The included reviews were not designed to isolate mediators, so mechanistic claims should remain provisional. Implementation also requires clinical nuance. Depression and anxiety can reduce motivation, energy, confidence, and attendance, and patients differ in physical comorbidity, baseline fitness, safety concerns, access to facilities, social preference, and interest in supervised, group-based, home-based, or digital programs. Public health guidance supports regular aerobic and muscle-strengthening activity for broad health benefits (40), but psychiatric translation requires individualized assessment, gradual progression, collaborative goal setting, adverse-event monitoring, adherence support, and coordination with ongoing pharmacotherapy or psychotherapy.

### Clinical implications

The present synthesis supports exercise primarily as an adjunctive or multimodal treatment component for depressive disorders rather than as a replacement for established evidence-based mental health care. Selected trials have compared exercise with pharmacotherapy or different exercise doses and contributed to the clinical appeal of exercise prescriptions (41, 42), but umbrella-level evidence does not justify a general claim that exercise is superior to psychotherapy or antidepressant medication. Clinically, exercise may be discussed as a patient-centered option aligned with symptom severity, treatment preference, comorbidity profile, physical-health goals, medication stability, access to services, and safety considerations. For anxiety disorders, clinicians should avoid overstating the evidence and should prioritize exercise as supportive care unless disorder-specific trial evidence and patient response justify a stronger role.

### Acceptability, tolerability, and safety reporting

Acceptability and safety should be interpreted independently from symptom efficacy. Similar attendance or dropout rates do not establish that exercise is well tolerated, because they do not necessarily capture symptom worsening, musculoskeletal injury, cardiovascular events, intervention burden, or reasons for discontinuation. Across the included reviews, adverse-event ascertainment was sparse and inconsistently reported, so the absence of a clear harms signal should be interpreted as insufficient evidence rather than evidence of no harm. Future studies should prespecify adverse-event definitions, systematically solicit harms, report adherence-adjusted exposure, and distinguish voluntary non-adherence from medically indicated discontinuation.

### Implications for future reviews and trials

Future reviews should predefine diagnostic boundaries, separate inactive from active and time-/attention-matched comparators, distinguish monotherapy from adjunctive exercise, identify primary overall estimates before subgroup analyses, and quantify overall and cluster-specific review overlap explicitly. This is important because redundant or weakly bounded reviews can inflate apparent certainty in clinical topics with many overlapping syntheses (43). Future trials should report exercise interventions in sufficient detail for replication, including frequency, intensity, time, type, progression, supervision, behavior-change support, co-interventions, medication stability, adherence thresholds, and deviations from prescription (44). Harms and discontinuation should be reported with the same rigor as benefits, using contemporary randomized-trial and harms-reporting standards (45, 46). For anxiety disorders in particular, trials should use disorder-specific measures and comparator designs that control for expectancy, time, attention, and social contact, and should avoid pooling generalized anxiety disorder, panic disorder, social anxiety disorder, and mixed anxiety presentations unless disorder-specific effects can also be examined.

### Strengths and limitations

Strengths include the strict focus on depressive and anxiety disorders, explicit separation of directly extractable pooled estimates from narrative-only evidence, diagnostic-rigor stratification, AMSTAR 2 appraisal, audit of effect-direction harmonization, primary-study overlap assessment, subset CCA analyses, and use of an evidence-overview plot to prevent overinterpretation of sparse outcomes. The main limitation is that umbrella conclusions remain constrained by the quality of the included reviews, most of which were low or critically low in confidence. Comparator frameworks, diagnostic definitions, intervention classes, and effect metrics differed materially across reviews, preventing defensible second-order pooling. Anxiety-disorder-specific and acceptability data were sparse, and adverse-event reporting was especially limited. Some modalities, including yoga, traditional Chinese exercise, and web-based exercise, were represented mainly by narrative, indirect, or non-directly comparable evidence. Finally, because eligibility was applied at the review level, some included reviews contained mixed or borderline populations that required conservative interpretation rather than complete exclusion; this issue was handled by diagnostic-rigor stratification and by not allowing indirect evidence to drive the principal diagnosed-disorder conclusions.

## Conclusion

Exercise interventions are supported most consistently for depressive symptom reduction in adults with formally diagnosed depressive disorders, particularly major depressive disorder. Anxiety-disorder-specific efficacy remains less secure and appears sensitive to comparator rigor, diagnostic composition, and localized overlap among anxiety-focused reviews. Acceptability outcomes do not currently indicate major differences from controls, but safety and tolerability reporting remain too sparse for confident conclusions. The most defensible clinical position is to frame exercise as a structured, individualized, and monitored adjunctive component of multimodal psychiatric care rather than as a replacement for established psychotherapy or pharmacotherapy.

## Data Availability

The original contributions presented in the study are included in the article/**Supplementary Material**. Further inquiries can be directed to the corresponding authors.
