# Supplementary material for: Exercise interventions are most consistently supported for depressive disorders: an umbrella review of diagnosed depressive and anxiety disorders
Source: Front Psychiatry. 2026 Jul 3;17:1871522. doi: 10.3389/fpsyt.2026.1871522 (PMC13376121; doi:10.3389/fpsyt.2026.1871522)
Supplement: Supplementary file 1 [file Supplementaryfile1.docx]

# Supplementary appendix

This supplementary file provides additional methods, extraction tables, AMSTAR 2 appraisal, primary-study overlap, subset CCA analyses, diagnostic-rigor stratification, evidence-direction audits, and non-core outcomes.

# Supplementary methods

The main-text quantitative figure was restricted to primary overall pooled estimates for core psychiatric symptom outcomes. Subgroup, sensitivity, publication-bias-adjusted, acceptability, and non-core physical or psychosocial outcomes were retained in supplementary tables.

For plotting only, effect directions were harmonized so that positive values favored exercise. Source-reported effect metrics and orientations were retained in extraction tables.

Corrected covered area was calculated from the normalized review-by-primary-study citation matrix. The auditable matrix contained 112 primary-study occurrences, 89 unique primary studies, and nine reviews, yielding CCA = (112 - 89) / (89 × 9 - 89) = 3.23%.

Diagnostic rigor was stratified to separate formally diagnosed evidence from indirect symptom-threshold or incompletely auditable review-level evidence. Core diagnosed-disorder interpretation required formal psychiatric diagnosis in all or nearly all primary studies, or documentation that at least 75% of included primary studies or participants involved formally diagnosed, treatment-seeking, or otherwise clearly clinical psychiatric samples. These judgments are summarized in Supplementary Table S16.

Subset CCA analyses were calculated to examine localized redundancy within the evidence clusters most relevant to the principal symptom conclusions. The core depression quantitative cluster, main quantitative anxiety estimates, broader anxiety symptom cluster, and anxiety-disorder-specific review subset are reported in Supplementary Table S17.

# Supplementary figure

**Supplementary Figure S1. Quantitative acceptability outcomes reported by included reviews.** Panel A displays attendance risk ratio, where values greater than 1 favor exercise. Panel B displays dropout risk difference, where values lower than 0 would favor exercise because fewer dropouts occurred in exercise. No umbrella-level pooling was performed because metrics differed and only two reviews reported pooled quantitative acceptability outcomes.


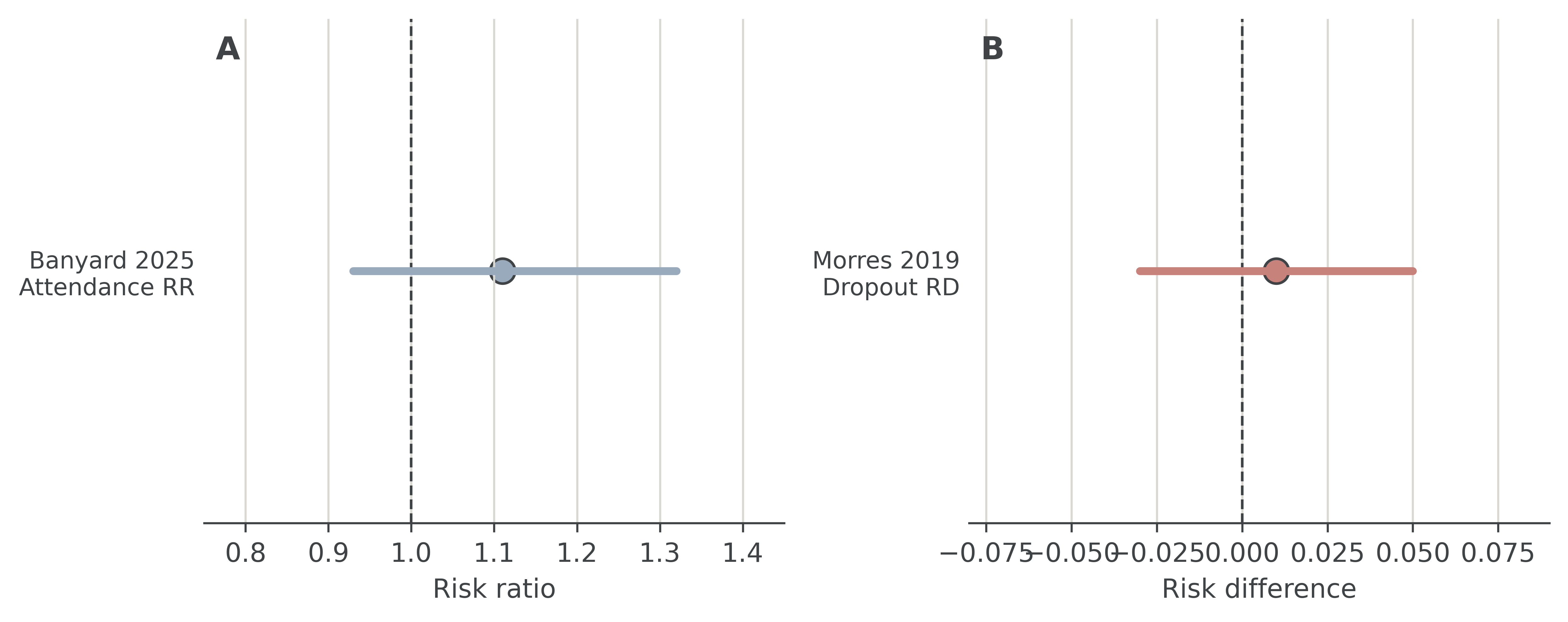


# Supplementary tables

## Supplementary Table S1. Search strategy.

| **Database** | **Strategy** |
| --- | --- |
| PubMed | ((Exercise[Mesh] OR Motor Activity[Mesh] OR Exercise Therapy[Mesh] OR Resistance Training[Mesh] OR Yoga[Mesh] OR Tai Ji[Mesh] OR Qigong[Mesh] OR exercise[Title/Abstract] OR physical activity[Title/Abstract] OR aerobic exercise[Title/Abstract] OR resistance training[Title/Abstract] OR yoga[Title/Abstract] OR tai chi[Title/Abstract] OR qigong[Title/Abstract] OR baduanjin[Title/Abstract] OR wuqinxi[Title/Abstract] OR web-based exercise[Title/Abstract]) AND (Depressive Disorder[Mesh] OR Depression[Mesh] OR Anxiety Disorders[Mesh] OR depress*[Title/Abstract] OR anxi*[Title/Abstract] OR major depressive disorder[Title/Abstract] OR panic disorder[Title/Abstract] OR social anxiety disorder[Title/Abstract]) AND (systematic review[Publication Type] OR meta-analysis[Publication Type] OR systematic review[Title/Abstract] OR meta-analysis[Title/Abstract] OR network meta-analysis[Title/Abstract])) |
| Web of Science | TS=(exercise OR physical activity OR aerobic exercise OR resistance training OR yoga OR tai chi OR qigong OR baduanjin OR wuqinxi OR web-based exercise) AND TS=(depress* OR anxi* OR major depressive disorder OR panic disorder OR social anxiety disorder OR dysthymi*) AND TS=(systematic review OR meta-analysis OR network meta-analysis) |
| SPORTDiscus | (TI/AB exercise OR physical activity OR aerobic exercise OR resistance training OR yoga OR tai chi OR qigong OR baduanjin OR wuqinxi OR web-based exercise) AND (TI/AB depress* OR anxi* OR major depressive disorder OR panic disorder OR social anxiety disorder OR dysthymi*) AND (TI/AB systematic review OR meta-analysis OR network meta-analysis) |
| Cochrane Library | #1 exercise OR physical activity OR aerobic exercise OR resistance training OR yoga OR tai chi OR qigong OR baduanjin OR wuqinxi OR web-based exercise:ti,ab,kw; #2 depress* OR anxi* OR major depressive disorder OR panic disorder OR social anxiety disorder:ti,ab,kw; #3 systematic review OR meta-analysis OR network meta-analysis:ti,ab,kw; #4 #1 AND #2 AND #3 |
| Embase | exp exercise/ OR exp motor activity/ OR exp exercise therapy/ OR exp resistance training/ OR exp yoga/ OR exp tai chi/ OR exp qigong/ OR exercise-related terms.ti,ab. AND exp depression/ OR exp depressive disorder/ OR exp anxiety disorder/ OR depression/anxiety terms.ti,ab. AND systematic review/meta-analysis terms.ti,ab. |
| Scopus | TITLE-ABS-KEY((exercise OR physical activity OR aerobic exercise OR resistance training OR yoga OR tai chi OR qigong OR baduanjin OR wuqinxi OR web-based exercise) AND (depress* OR anxi* OR major depressive disorder OR panic disorder OR social anxiety disorder) AND (systematic review OR meta-analysis OR network meta-analysis)) |

## Supplementary Table S2. Review-reported search methods.

| **Review** | **Databases searched** | **Search end date** | **Language limits** | **Supplementary searching** |
| --- | --- | --- | --- | --- |
| Banyard 2025 | EBSCOhost, Scopus, Web of Science, PsychNET, PubMed | Through 24 Feb 2024 | English only | Reference-list searching |
| Bartley 2013 | PubMed, PsycINFO | Through Jan 2013 | No language restrictions reported | Reference lists |
| Carneiro 2022 | Cochrane Library, EBSCO, PubMed, SciELO, Scopus, Web of Science | 18 Feb 2021 | No language/date limits declared | Reference lists, expert consultation, errata/retraction search |
| Cramer 2017 | MEDLINE/PubMed, Scopus, CENTRAL | 6 Dec 2016 | No language restrictions | Own database, manual searching, reference lists |
| Feng 2024 | PubMed, Cochrane Library, Web of Science, CNKI, VIP, CBM, Wan Fang | Jul 2023 | Chinese and English | Not explicitly beyond database search |
| Jayakody 2014 | MEDLINE, EMBASE, PsycINFO, CINAHL, AMED, Cochrane | Jul 2011 | Not clear | Hand-searching, bibliography checks, author contact |
| Machado 2022 | PubMed, ISI Web of Science, CENTRAL | Mar 2021 | English only | Manual screening, expert suggestions, author contact |
| Morres 2019 | Scopus, PubMed, PEDro, PsycINFO, SPORTDiscus, Academic Search Complete, ERIC, ProQuest, TRoPHI, ClinicalTrials.gov, WHO ICTRP | 1980 to Mar 2017 | English only | Bibliographies of 19 systematic reviews |
| Seshadri 2021 | Ovid MEDLINE, EMBASE, PsycINFO, Cochrane | Mar 2019; updated 4 Nov 2019 | English-language RCTs | Update noted |

## Supplementary Table S3. Updated AMSTAR 2 item-level appraisal.

| **Study** | **Q1** | **Q2** | **Q3** | **Q4** | **Q5** | **Q6** | **Q7** | **Q8** | **Q9** | **Q10** | **Q11** | **Q12** | **Q13** | **Q14** | **Q15** | **Q16** | **Quality** |
| --- | --- | --- | --- | --- | --- | --- | --- | --- | --- | --- | --- | --- | --- | --- | --- | --- | --- |
| Banyard 2025 | Y | PY | Y | PY | Y | N | N | Y | Y | N | Y | N | Y | Y | Y | Y | Low |
| Bartley 2013 | Y | N | Y | PY | N | N | N | PY | N | N | Y | N | N | Y | Y | Y | Critically low |
| Carneiro 2022 | Y | Y | Y | Y | Y | Y | Y | Y | Y | Y | NA | NA | Y | Y | NA | Y | High |
| Cramer 2017 | Y | N | Y | PY | Y | Y | Y | Y | Y | N | NA | NA | Y | Y | NA | Y | Low |
| Feng 2024 | Y | Y | Y | PY | Y | Y | N | PY | Y | N | Y | N | Y | Y | Y | Y | Low |
| Jayakody 2014 | Y | N | Y | PY | Y | N | N | Y | Y | N | NA | NA | Y | Y | NA | Y | Critically low |
| Machado 2022 | Y | N | Y | PY | Y | Y | N | Y | Y | N | NA | NA | Y | Y | NA | Y | Critically low |
| Morres 2019 | Y | N | Y | PY | Y | Y | N | PY | Y | N | Y | Y | Y | Y | Y | N | Critically low |
| Seshadri 2021 | Y | N | Y | PY | Y | Y | N | Y | Y | N | Y | Y | Y | Y | Y | Y | Critically low |

## Supplementary Table S4. Master extraction of review-level quantitative effects.

| **Review** | **Estimate class** | **Disorder domain** | **Intervention** | **Comparator** | **Outcome** | **Metric** | **Effect** | **CI lower** | **CI upper** | **I²** |
| --- | --- | --- | --- | --- | --- | --- | --- | --- | --- | --- |
| Morres 2019 | Primary overall | MDD | Aerobic exercise | Non-exercise | Depression severity | Hedges g | -0.79 | -1.00 | -0.57 | 21.0 |
| Morres 2019 | Acceptability | MDD | Aerobic exercise | Non-exercise | Dropout risk difference | RD | 0.01 | -0.03 | 0.05 | 0.0 |
| Seshadri 2021 | Primary overall | MDD outpatient | Exercise/yoga/tai chi | All controls | Depression severity | Hedges g | 0.63 | 0.50 | 0.76 | 98.0 |
| Seshadri 2021 | Trim-and-fill | MDD outpatient | Exercise/yoga/tai chi | All controls | Depression severity | Hedges g | 0.11 | -0.03 | 0.26 |  |
| Banyard 2025 | Primary overall | Diagnosed depression/anxiety | Aerobic/resistance/mixed | All controls | Depression symptoms | SMD | -0.97 | -1.28 | -0.66 | 90.1 |
| Banyard 2025 | Primary overall | Diagnosed depression/anxiety | Aerobic/resistance/mixed | All controls | Anxiety symptoms | SMD | -0.66 | -1.09 | -0.23 | 85.8 |
| Banyard 2025 | Acceptability | Diagnosed depression/anxiety | Aerobic/resistance/mixed | Controls | Attendance | RR | 1.11 | 0.93 | 1.32 | 35.9 |
| Banyard 2025 | Non-core secondary | Diagnosed depression/anxiety | Aerobic/resistance/mixed | All comparators | 6-min walk test | SMD | 3.39 | 2.48 | 4.30 | 0.0 |
| Banyard 2025 | Non-core secondary | Diagnosed depression/anxiety | Aerobic/resistance/mixed | All comparators | VO2 max | SMD | 0.40 | -0.35 | 1.10 | 88.7 |
| Banyard 2025 | Non-core secondary | Diagnosed depression/anxiety | Aerobic/resistance/mixed | All comparators | Self-efficacy | SMD | 0.35 | -0.37 | 1.08 | 68.4 |
| Bartley 2013 | Primary overall | DSM-IV anxiety disorders | Aerobic exercise | Selected controls | Anxiety symptoms | SMD | 0.02 | -0.20 | 0.24 |  |
| Bartley 2013 | Medication comparison | DSM-IV anxiety disorders | Aerobic exercise | Antidepressant medication | Anxiety symptoms | SMD | -0.28 | -0.76 | 0.20 |  |

## Supplementary Table S5. Harmonized quantitative effects used for cross-review plotting.

| **Review** | **Outcome** | **Metric** | **Original orientation** | **Harmonization rule** | **Plotted estimate** | **Plotted 95% CI** | **Figure usage** |
| --- | --- | --- | --- | --- | --- | --- | --- |
| Morres 2019 | Depression severity | Hedges g | Negative values favor exercise | Multiply by -1 | 0.79 | 0.57 to 1.00 | Main Figure 4 |
| Seshadri 2021 | Depression severity | Hedges g | Positive values shown as benefit | No sign change | 0.63 | 0.50 to 0.76 | Main Figure 4 |
| Banyard 2025 | Depression symptoms | SMD | Negative values favor exercise | Multiply by -1 | 0.97 | 0.66 to 1.28 | Main Figure 4 |
| Banyard 2025 | Anxiety symptoms | SMD | Negative values favor exercise | Multiply by -1 | 0.66 | 0.23 to 1.09 | Main Figure 4 |
| Bartley 2013 | Anxiety symptoms | SMD | Source estimate retained | No sign change | 0.02 | -0.20 to 0.24 | Main Figure 4 |

## Supplementary Table S6. Narrative findings extracted where no directly pooled main effect was available.

| **Review** | **Disorder domain** | **Intervention** | **Summary finding** | **Key caveat** |
| --- | --- | --- | --- | --- |
| Cramer 2017 | MDD | Yoga | Some evidence beyond placebo; no firm recommendation possible | Few small heterogeneous trials |
| Jayakody 2014 | Clinical anxiety disorders | Exercise/adjunctive exercise | Adjunctive benefit may occur in selected comparisons | Mostly single-study findings |
| Machado 2022 | Panic disorder | Regular exercise | No clear panic-symptom reduction; global anxiety/depression often improved | Heterogeneous trials |
| Carneiro 2022 | Depressive/anxiety disorders or above-threshold symptoms | Web-based exercise | No clear anxiety superiority; one study found depressive benefit | Only 3 RCTs |
| Feng 2024 | Depression | Traditional Chinese exercise | Authors concluded Baduanjin/Tai Chi/Wuqinxi were effective | Direction/orientation not fully auditable |

## Supplementary Table S7. Publication-bias, influence, and sensitivity findings.

| **Review** | **Domain** | **Finding** | **Interpretation** |
| --- | --- | --- | --- |
| Seshadri 2021 | Publication bias | Trim-and-fill reduced g = 0.63 to g = 0.11 (-0.03 to 0.26) | Most consequential fragility signal |
| Seshadri 2021 | Sensitivity | Effect attenuated after outlier removal and restriction to lower-risk studies | Magnitude uncertain |
| Morres 2019 | Publication bias | No publication bias detected in primary synthesis | Supports relative confidence but does not remove AMSTAR concerns |
| Banyard 2025 | Influence/small-study effects | No single-study dominance; funnel-plot/small-study concerns noted | High heterogeneity remains important |
| Bartley 2013 | Comparator moderation | Large apparent benefits limited to waitlist/placebo or non-time-controlled trials | Comparator rigor materially influenced effect size |

## Supplementary Table S8. Acceptability and tolerability findings.

| **Population** | **Intervention** | **Outcome** | **Review(s)** | **Result** | **95% CI/heterogeneity** | **Summary note** |
| --- | --- | --- | --- | --- | --- | --- |
| Diagnosed depression and/or anxiety | Aerobic/resistance/mixed exercise | Attendance | Banyard 2025 | RR = 1.11 | 0.93 to 1.32; I² = 35.9% | Similar attendance; no clear acceptability advantage. |
| MDD | Aerobic exercise | Dropout risk difference | Morres 2019 | RD = 0.01 | -0.03 to 0.05; I² = 0% | No meaningful dropout difference. |
| Depressive/anxiety disorders or above-threshold symptoms | Web-based exercise | Compliance/dropout | Carneiro 2022 | Narrative | — | Compliance low and dropout notable in a small evidence base. |
| MDD | Yoga | Treatment-related adverse events | Cramer 2017 | Narrative | — | Only two RCTs assessed treatment-related adverse events; none reported treatment-related adverse events. |
| Clinical anxiety disorders/panic disorder | Exercise | Compliance/dropout | Jayakody 2014; Machado 2022 | Narrative | — | Sparse and non-pooled reporting; no robust acceptability conclusion. |

## Supplementary Table S9. Review-pairwise overlap long-form table.

| **Review A** | **Review B** | **Shared primary-study entries** |
| --- | --- | --- |
| Banyard 2025 | Bartley 2013 | 1 |
| Banyard 2025 | Carneiro 2022 | 1 |
| Banyard 2025 | Jayakody 2014 | 2 |
| Banyard 2025 | Machado 2022 | 1 |
| Banyard 2025 | Morres 2019 | 2 |
| Banyard 2025 | Seshadri 2021 | 6 |
| Bartley 2013 | Jayakody 2014 | 4 |
| Bartley 2013 | Machado 2022 | 4 |
| Cramer 2017 | Seshadri 2021 | 2 |
| Feng 2024 | Seshadri 2021 | 2 |
| Jayakody 2014 | Machado 2022 | 3 |
| Morres 2019 | Seshadri 2021 | 2 |

## Supplementary Table S10. Pairwise overlap matrix of primary-study entries across included reviews.

| **Review** | **Banyard 2025** | **Bartley 2013** | **Carneiro 2022** | **Cramer 2017** | **Feng 2024** | **Jayakody 2014** | **Machado 2022** | **Morres 2019** | **Seshadri 2021** |
| --- | --- | --- | --- | --- | --- | --- | --- | --- | --- |
| Banyard 2025 | 30 | 1 | 1 | 0 | 0 | 2 | 1 | 2 | 6 |
| Bartley 2013 | 1 | 7 | 0 | 0 | 0 | 4 | 4 | 0 | 0 |
| Carneiro 2022 | 1 | 0 | 1 | 0 | 0 | 0 | 0 | 0 | 0 |
| Cramer 2017 | 0 | 0 | 0 | 7 | 0 | 0 | 0 | 0 | 2 |
| Feng 2024 | 0 | 0 | 0 | 0 | 16 | 0 | 0 | 0 | 2 |
| Jayakody 2014 | 2 | 4 | 0 | 0 | 0 | 8 | 3 | 0 | 0 |
| Machado 2022 | 1 | 4 | 0 | 0 | 0 | 3 | 7 | 0 | 0 |
| Morres 2019 | 2 | 0 | 0 | 0 | 0 | 0 | 0 | 11 | 2 |
| Seshadri 2021 | 6 | 0 | 0 | 2 | 2 | 0 | 0 | 2 | 25 |

Overall CCA = 3.23%, based on 112 primary-study occurrences, 89 unique primary studies, and nine reviews.

## Supplementary Table S11. Eligibility and boundary audit for the main efficacy figure.

| **Quantitative row** | **Status** | **Reason** |
| --- | --- | --- |
| Morres 2019 primary overall | Eligible | Primary overall pooled estimate for core psychiatric symptom outcome |
| Seshadri 2021 primary overall | Eligible | Primary overall pooled estimate for core psychiatric symptom outcome |
| Seshadri 2021 trim-and-fill | Supplementary only | Publication-bias-adjusted sensitivity estimate |
| Banyard 2025 depression | Eligible | Primary overall pooled estimate |
| Banyard 2025 anxiety | Eligible | Primary overall pooled estimate |
| Banyard 2025 attendance | Supplementary only | Acceptability outcome |
| Banyard 2025 6MWT/VO2 max/self-efficacy | Supplementary only | Non-core secondary outcomes |
| Bartley 2013 primary overall | Eligible | Primary overall pooled estimate for anxiety symptoms |
| Bartley 2013 medication comparison | Supplementary only | Direct comparator subgroup |
| Carneiro/Cramer/Feng/Jayakody/Machado | Narrative only | No directly comparable pooled overall estimate or orientation not fully auditable |

## Supplementary Table S12. Direction harmonization audit.

| **Review** | **Metric** | **Outcome** | **Original orientation** | **Final plotting rule** |
| --- | --- | --- | --- | --- |
| Morres 2019 | Hedges g | Depression severity | Negative values favor exercise | Multiply by -1 |
| Seshadri 2021 | Hedges g | Depression severity | Positive values shown as benefit | No sign change |
| Banyard 2025 | SMD | Depression symptoms | Negative values favor exercise | Multiply by -1 |
| Banyard 2025 | SMD | Anxiety symptoms | Negative values favor exercise | Multiply by -1 |
| Bartley 2013 | SMD | Anxiety symptoms | Source estimate retained | No sign change |

## Supplementary Table S13. Direct comparator evidence against pharmacotherapy and psychotherapy.

| **Review** | **Domain** | **Exercise** | **Comparator** | **Outcome** | **Best available result** | **Key caveat** |
| --- | --- | --- | --- | --- | --- | --- |
| Morres 2019 | MDD | Aerobic exercise | Antidepressants or TAU | Depression severity | Hedges g = -0.75 (-1.01 to -0.48) | Not a pure medication-only comparison |
| Morres 2019 | MDD | Aerobic exercise | Psychological treatments | Depression severity | Hedges g = -0.85 (-1.21 to -0.48) | Comparator subgroup rather than psychotherapy-only meta-analysis |
| Bartley 2013 | DSM-IV anxiety disorders | Aerobic exercise | Antidepressant medication | Anxiety symptoms | SMD = -0.28 (-0.76 to 0.20) | Small evidence base; no superiority |
| Cramer 2017 | MDD | Yoga | Imipramine | Depression | No significant short-term difference reported | Single small trial |
| Cramer 2017 | MDD | Yoga | ECT | Depression/remission | ECT favored | Single small comparison |
| Jayakody 2014 | Panic disorder | Exercise | Antidepressant medication | Anxiety | Medication favored | Single-study evidence |
| Machado 2022 | Panic disorder | Regular exercise | Psychotherapy/pharmacotherapy/controls | Panic/global anxiety/depression | Mixed study-level findings | No pooled head-to-head synthesis |

## Supplementary Table S14. Non-core secondary outcomes reported outside the main text.

| **Review** | **Domain** | **Outcome** | **Metric/effect** | **95% CI** | **Heterogeneity** | **Interpretation** |
| --- | --- | --- | --- | --- | --- | --- |
| Banyard 2025 | Diagnosed depression/anxiety | 6-min walk test | SMD = 3.39 | 2.48 to 4.30 | I² = 0% | Favours exercise; non-core physical function outcome |
| Banyard 2025 | Diagnosed depression/anxiety | VO2 max | SMD = 0.40 | -0.35 to 1.10 | I² = 88.7% | Imprecise and heterogeneous |
| Banyard 2025 | Diagnosed depression/anxiety | Self-efficacy | SMD = 0.35 | -0.37 to 1.08 | I² = 68.4% | Imprecise and heterogeneous |

## Supplementary Table S15. Evidence-overview data used for Figure 5.

| **Outcome** | **Outcome_Order** | **Evidence_Direction** | **Evidence_Y** | **Contributing_Reviews** | **Credibility** | **Credibility_Group** | **Short_Label** |
| --- | --- | --- | --- | --- | --- | --- | --- |
| Depression symptom severity | 1 | Generally favorable | 3 | 7 | Low | Low | Depression |
| Anxiety symptom severity | 2 | Mixed / inconsistent | 2 | 7 | Low | Low | Anxiety |
| Remission / response | 3 | No clear conclusion | 1 | 1 | Very low | Very low | Remission |
| Attendance / adherence | 4 | No clear difference / sparse | 1 | 2 | Very low | Very low | Attendance |
| Dropout / discontinuation | 5 | No clear difference / sparse | 1 | 4 | Very low | Very low | Dropout |
| Adverse events / tolerability | 6 | Too sparse to judge | 0 | 2 | Very low | Very low | Adverse events |
| 6-min walk test | 7 | Generally favorable | 3 | 1 | Very low | Very low | 6MWT |
| VO2 max | 8 | Mixed / imprecise | 2 | 1 | Very low | Very low | VO2 max |
| Self-efficacy | 9 | Mixed / imprecise | 2 | 1 | Very low | Very low | Self-efficacy |

## Supplementary Table S16. Diagnostic-rigor and indirectness stratification of included reviews.

| **Review** | **Disorder/domain represented** | **Diagnostic-rigor stratum** | **Formal-diagnosis requirement from review eligibility** | **Mixed psychiatric-medical eligibility** | **Role in synthesis / indirectness handling** |
| --- | --- | --- | --- | --- | --- |
| Banyard 2025 | Diagnosed depression and/or anxiety | High for review-level eligibility | Adults 18–64 years with diagnosed depression and/or anxiety. | Eligible only as diagnosed psychiatric samples; not treated as medical-comorbidity evidence. | Core mixed diagnosed depression/anxiety quantitative estimates; magnitude qualified by heterogeneity. |
| Bartley 2013 | DSM-IV anxiety disorders | High | Primary DSM-defined anxiety disorder; PTSD excluded. | Not a medical-mixed review. | Core anxiety-disorder-specific pooled estimate. |
| Carneiro 2022 | Depressive/anxiety disorders or above-threshold symptoms | Indirect / low-to-moderate | DSM/ICD diagnosis or validated symptom thresholds; formal-diagnosis proportion not consistently auditable. | Mixed diagnostic/symptom-threshold boundary; not used as medical-comorbidity evidence. | Narrative only; not used to drive principal diagnosed-disorder quantitative conclusions. |
| Cramer 2017 | Major depressive disorder | Moderate-to-high | DSM-IV/V MDD; post-hoc inclusion if ≥75% MDD. | Not a medical-mixed review. | Narrative MDD yoga evidence. |
| Feng 2024 | Depression | High | Adults meeting diagnostic criteria for depression. | Not primarily medical-mixed. | Narrative/network evidence retained as supplementary because league-table orientation was not sufficiently auditable for the main quantitative hierarchy. |
| Jayakody 2014 | Clinical anxiety disorders | Moderate-to-high | Formal ICD/DSM or validated anxiety-disorder diagnosis. | Not primarily medical-mixed. | Narrative anxiety-disorder evidence; mostly single-study comparisons. |
| Machado 2022 | Panic disorder | High | DSM/ICD/MINI panic disorder with or without agoraphobia. | Not medical-mixed. | Panic-disorder-specific narrative evidence. |
| Morres 2019 | Major depressive disorder | High | Clinical MDD diagnosis; recruited through mental health services. | Psychiatric-service samples; not medical-mixed. | Core depression quantitative estimate. |
| Seshadri 2021 | Outpatient major depressive disorder | High | Current-episode MDD, ICD-10 or DSM-IV/5, with ≥8 weeks follow-up. | Outpatient MDD target; not medical-mixed. | Core depression quantitative estimate; interpreted cautiously because of extreme heterogeneity and publication-bias sensitivity. |

Note: Core diagnosed-disorder interpretation required formal psychiatric diagnosis in all or nearly all primary studies, or documentation that at least 75% of included primary studies or participants were formally diagnosed, treatment-seeking, or otherwise clearly clinical psychiatric samples. Reviews that did not meet or did not clearly report this threshold were retained only as indirect narrative or supplementary evidence.

## Supplementary Table S17. Subset corrected covered area analyses by evidence cluster.

| **Evidence cluster** | **Reviews included** | **Occurrences (N)** | **Unique studies (r)** | **Reviews (c)** | **CCA** | **Interpretation** | **Use in interpretation** |
| --- | --- | --- | --- | --- | --- | --- | --- |
| Overall normalized matrix | All nine included reviews | 112 | 89 | 9 | 3.23% | Slight | Describes broad review ecosystem. |
| Core depression quantitative cluster | Morres 2019; Seshadri 2021; Banyard 2025 | 66 | 58 | 3 | 6.90% | Moderate | Qualifies depression effect estimates; no second-order pooling performed. |
| Broader depression-relevant cluster | Cramer 2017; Feng 2024; Morres 2019; Seshadri 2021; Banyard 2025; Carneiro 2022 | 90 | 77 | 6 | 3.38% | Slight | Shows that adding narrative/supplementary depression-related reviews dilutes overlap. |
| Main quantitative anxiety estimates | Bartley 2013; Banyard 2025 | 37 | 36 | 2 | 2.78% | Slight | Directly relevant to the two plotted anxiety rows in Figure 4. |
| Core anxiety symptom cluster | Bartley 2013; Jayakody 2014; Machado 2022; Banyard 2025 | 52 | 42 | 4 | 7.94% | Moderate | Qualifies anxiety conclusions because related reviews share primary trials. |
| Anxiety-disorder-specific reviews | Bartley 2013; Jayakody 2014; Machado 2022 | 22 | 14 | 3 | 28.57% | Very high | Sensitivity analysis showing localized redundancy within anxiety-specific review evidence. |

CCA interpretation followed conventional categories: 0–5% slight, 6–10% moderate, 11–15% high, and >15% very high overlap. Values were calculated from the same normalized review-by-primary-study matrix used for the overall CCA.
